# Supplementary material for: Quantifying patterns of alcohol consumption and its effects on health and wellbeing among BaYaka hunter-gatherers: A mixed-methods cross-sectional study
Source: PLoS One. 2021 Oct 27;16(10):e0258384. doi: 10.1371/journal.pone.0258384 (PMC8550590; doi:10.1371/journal.pone.0258384)
Supplement: S1 File — (DOCX) [file pone.0258384.s001.docx]

**Supporting Information**

**S1 Table. English translations of questions asked in structured interviews**

| **Theme** | **Question** |
| --- | --- |
| Alcohol preferences | 1. What is your favourite alcoholic drink? |
| Access to alcohol | 2a. Where did you get the alcohol from?  2b. [if bought] Where did you get the money?  3a. [Longa only] Do you ever enter debt with the Bantu people?  3b. [if yes] Is there interest? |
| Reasons for drinking alcohol | 4. Why do you drink alcohol? |
| Alcohol and maternal/child health | 5. Did you/your wife [as appropriate] drink alcohol whilst pregnant?  6. Did you/your wife drink alcohol whilst breastfeeding? |
| Problems with alcohol | 7. Do you think drinking alcohol causes problems? |

**S2 Table. Results of multiple regression models of the effects of age and sex on alcohol consumption behaviours**

| **Dependent variable** | **Predictors** | **Coefficient** | **Standard error** | **P value** | **Adjusted R-squared** |
| --- | --- | --- | --- | --- | --- |
| **Frequency of weekly alcohol consumption (times/week)** | Sex (M) | 0.22413 | 0.49078 | 0.65 | 0.1374 |
|  | Age | 0.04669 | 0.01361 | 0.0011 |  |
| **Total weekly alcohol consumption (cups)** | Sex (M) | 4.588098 | 2.076143 | 0.031 | 0.1603 |
|  | Age | 0.677338 | 0.288842 | 0.022 |  |
|  | Age squared | -0.005947 | 0.003045 | 0.055 |  |
| **Amount of alcohol consumed per time (cups)** | Sex (M) | 1.643687 | 0.527084 | 0.0027 | 0.1823 |
|  | Age | 0.146021 | 0.073878 | 0.052 |  |
|  | Age squared | -0.001314 | 0.000779 | 0.097 |  |

**S3 Table. Results of multiple regression models of effects of alcohol consumption on physiological variables**

| **Dependent variable** | **Predictors** | **Coefficient** | **Standard error** | **P value** | **Adjusted R-squared** |
| --- | --- | --- | --- | --- | --- |
| **Systolic blood pressure (mmHg)** | Total weekly alcohol consumption | 1.97735 | 0.86388 | 0.026 | 0.2372 |
|  | Age | 0.62646 | 0.15722 | 0.00019 |  |
|  | Sex (M) | 2.86209 | 5.32051 | 0.59 |  |
|  | Total weekly alcohol consumption * sex (M) | -0.73986 | 0.48096 | 0.13 |  |
|  | Total weekly alcohol consumption * age | -0.03105 | 0.01620 | 0.060 |  |
| **Diastolic blood pressure (mmHg)** | Total weekly alcohol consumption | 0.756532 | 0.397273 | 0.062 | 0.2427 |
|  | Age | 0.261696 | 0.089345 | 0.0048 |  |
|  | Sex (M) | -8.02129 | 2.208248 | 0.00045 |  |
|  | Total weekly alcohol consumption * age | -0.013246 | 0.008908 | 0.14 |  |
| **Haemoglobin (g/L)** | Total weekly alcohol consumption | -0.08597 | 0.46377 | 0.85 | 0.3339 |
|  | Age | -0.05007 | 0.08528 | 0.56 |  |
|  | Sex (M) | 10.06671 | 4.47346 | 0.029 |  |
|  | Total weekly alcohol consumption * sex (M) | 0.52446 | 0.50573 | 0.30 |  |
| **White blood cell count** | Total weekly alcohol consumption | -0.140222 | 0.148874 | 0.35 | -0.00596 |
|  | Age | -0.146917 | 0.106866 | 0.18 |  |
|  | Sex (M) | 0.347146 | 0.788518 | 0.66 |  |
|  | Total weekly alcohol consumption * sex (M) | 0.001179 | 0.001090 | 0.29 |  |
|  | Total weekly alcohol consumption * age | 0.004843 | 0.003433 | 0.17 |  |
| **Glycated haemoglobin** | Total weekly alcohol consumption | 0.494415 | 0.291997 | 0.010 | 0.1762 |
|  | Age | 0.463346 | 0.190856 | 0.019 |  |
|  | Sex (M) | -0.194366 | 1.445010 | 0.89 |  |
|  | Age squared | -0.004472 | 0.002006 | 0.031 |  |
|  | Total weekly alcohol consumption * age | -0.007116 | 0.006440 | 0.27 |  |
| **Body mass index** | Total weekly alcohol consumption | -0.0811726 | 0.0579735 | 0.17 | 0.06985 |
|  | Age | 0.1540682 | 0.0790736 | 0.056 |  |
|  | Sex (M) | -1.0441744 | 0.7761367 | 0.18 |  |
|  | Age squared | -0.0018583 | 0.0008162 | 0.027 |  |
|  | Total weekly alcohol consumption * sex | 0.1034175 | 0.0673884 | 0.13 |  |
| **Heart rate (bpm)** | Total weekly alcohol consumption | 0.4192 | 0.4155 | 0.32 | 0.09267 |
|  | Age | -0.1099 | 0.1151 | 0.34 |  |
|  | Sex (M) | -11.5607 | 5.6308 | 0.044 |  |
|  | Total weekly alcohol consumption * sex (M) | -0.1511 | 0.4940 | 0.76 |  |
|  |  |  |  |  |  |
| **Dependent variable** | **Predictors** | **Coefficient (odds ratio)** | **Standard error** | **P value** | **Pseudo R-squared** |
| **Reported diarrhoea** | Total weekly alcohol consumption | 0.108893  (1.115043) | 0.041222 | 0.00825 | 0.1264418 |
|  | Age | 0.002599  (1.002602) | 0.015627 | 0.86791 |  |
|  | Sex (M) | -1.048888  (0.350327) | 0.630729 | 0.09632 |  |
| **Reported cough** | Total weekly alcohol consumption | 0.06843  (1.070826) | 0.05047 | 0.175 | 0.1099956 |
|  | Age | 0.03203  (1.032548) | 0.01996 | 0.109 |  |
|  | Sex (M) | 0.05820  (1.059927) | 0.61402 | 0.924 |  |

**S4 Table. Responses to question about whether alcohol causes problems, separated by camp**

| **Effect of alcohol** | **Sembola, n=32 (percentage)** | **Longa, n=28 (percentage)** | **Total, n=54 (percentage)** |
| --- | --- | --- | --- |
| Violence (unspecified) | 53.1 | 31.8 | 44.4 |
| Domestic violence | 12.5 | 0.0 | 7.4 |
| Disorder/disturbance | 9.4 | 18.2 | 13.0 |
| No problems | 18.8 | 4.5 | 13.0 |
| Good during music/dance | 0.0 | 27.3 | 11.1 |
| Talking nonsense | 3.1 | 13.6 | 7.4 |
| Health problems | 3.1 | 0.0 | 1.9 |
| Good for mood | 0.0 | 4.5 | 1.9 |
| **Total** | **100** | **100** | **100** |

Coding categories for responses to question about reasons for drinking:

- Mood alteration – responses that mentioned using alcohol to change mood, including those describing positive mood after using alcohol (e.g. “joy”, “courage”, “to have fun” or to relax) as well as those suggesting that drinking was used to reduce negative emotions (e.g. “get rid of bad thoughts”, “stay sane”, “thinking/worrying [unhappy] too much”, “get rid of *mawa*” [Mbendjele: ‘sorrow’]).
- Habit or need – responses which indicated that the interviewee felt compelled to consume alcohol, or that consuming alcohol is a regular habit. Several people said they bought alcohol as soon as they were paid; three that alcohol was “like food”; and one that they were unable to stop drinking alcohol. Others mentioned habituation from an early age, often being given alcohol by a family member.
- Benefit to body – responses that indicated a physical benefit from alcohol consumption, often ambiguous (e.g. general idea of ‘encouraging’ the body) but occasionally specific (e.g. that alcohol contains ‘vitamins’ or can get rid of a cold).
- Social drinking – responses indicating that alcohol consumption is conditional on other people. This included drinking to relax with friends (e.g. “share when cooking”, “nice to have with others”), as well as drinking due to peer pressure. Social alcohol use during music and ritual is coded separately.
- Ritual/song and dance – responses which indicated that alcohol was used when singing, dancing, or during ritual events. Mbendjele ritual is characteristically based on polyphonic singing and dance (8).

Coding categories for responses to question about problems caused by alcohol:

- No problems – responses specifying that alcohol is not problematic.
- Health problems – responses indicating that alcohol can cause physical health issues
- Violence, unspecified – responses linking alcohol to violence of an unspecified nature or direction. People most commonly simply said “fights”, but some also mentioned unprovoked aggression after drinking, and drunk people provoking others.
- Domestic violence – responses specifically linking alcohol to violence towards wives (e.g. “fight with/beat wife”).
- Disorder/disturbance – responses linking alcohol to non-specific disorderly behaviour after alcohol use. Frequently people simply said “disorder”.
- Talking nonsense - responses linking alcohol use to silly, provocative or nonsensical speaking
- Good during music/dance – responses describing positive effects of drinking during music or dancing, including specifically ritual use. Interviewees said that alcohol means “everyone dances well”, and that drinking is good with music.
- Good for mood – responses describing positive effects of alcohol use on emotional state, such as getting rid of tiredness after work.
